# Frontal sinus infection leading to sino-orbital aspergillosis: a case report

**DOI:** 10.11604/pamj.2021.40.95.28261

**Published:** 2021-10-13

**Authors:** Camille Yvon, Didar Abdulla, Sarah Watson, Izhar Bagwan, Christopher Mclean

**Affiliations:** 1 Royal Surrey County Hospital, Guildford, United Kingdom

**Keywords:** Aspergilloma, fungal disease, frontal sinus, voriconazole, case report

## Abstract

Sino-orbital aspergillosis is an uncommon but aggressive infection. It rarely originates from the frontal sinus due to the complex anatomy of the frontal recess and anteromedial position of its ostium. An 87-year-old man of Nigerian heritage with a history of multiple myeloma, chronic kidney disease and type 2 diabetes, presented to the eye clinic with a right tense swollen eyelid and proptosis. Computed tomography (CT) and magnetic resonance imaging (MRI) scan revealed a right superomedial mass communicating with the frontal sinus and biopsy confirmed an orbital aspergilloma. The patient was successfully treated with debulking surgery and anti-fungal treatment despite developing side effects to the drugs. To improve prognosis, ophthalmologists should be aware of this distinct entity and use a multi-disciplinary approach.

## Introduction

Sino-orbital aspergillosis is a rare condition that is caused by an infection of the paranasal sinuses spreading to the orbit. It can be divided into: invasive (causing bony erosion, hematologic infections; and commonly affects the immunocompromised) and non-invasive types [1]. Diagnosis can be challenging due to the masquerading signs and often difficult endoscopic access. Sino-orbital aspergillosis rarely originates from the frontal sinus due to the variable anatomy of the frontal recess and position of its sinus outflow tract. We describe a rare case of sino-orbital aspergillosis likely arising from the frontal sinus in a patient with a background of multiple myeloma.

## Patient and observation

**Patient information:** an 87-year-old Nigerian businessman presented to the eye clinic with a 3-week history of right orbital swelling. He divided his time between the United Kingdom and rural Nigeria. He had longstanding myeloma with previous bone marrow transplant in 2002 and had been on thalidomide derivative therapy for many years. He also had a background of chronic kidney disease (CKD) and well controlled type 2 diabetes mellitus (T2DM).

**Figure 1:**
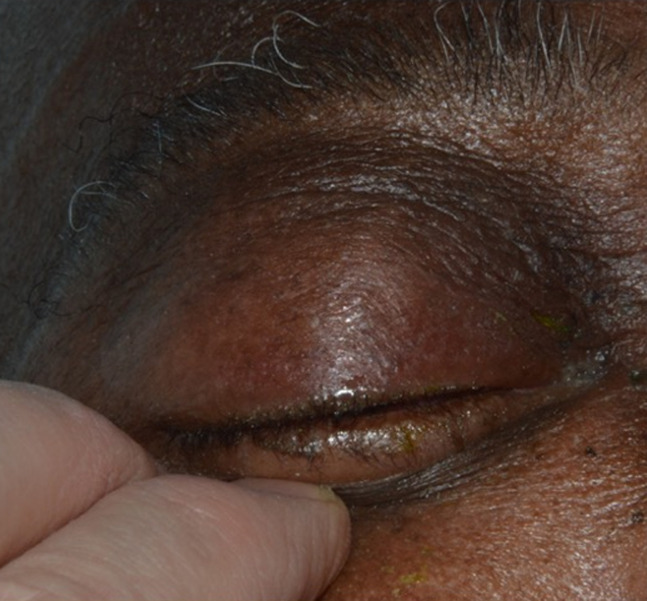
photograph of external eye demonstrating swelling and complete drooping of the right eyelid

**Clinical findings:** on examination, he had slightly reduced visual acuities (6/12 right eye, 6/9 left eye) with no relative afferent pupillary defect, and known deuteranomaly. The right eyelid was very swollen and erythematous; his lids had to be pulled open for examination (Figure 1). There appeared to be proptosis, significant conjunctival chemosis, and restricted eye movements. Anterior segment and fundoscopy examination were unremarkable. He was apyrexic and was noted to have weight loss at a routine oncology appointment.

**Timeline of current episode:** initial diagnosis was presumed orbital cellulitis; this did not respond to antibiotics started in primary care. He subsequently underwent further investigations on hospital admission.

**Figure 2:**
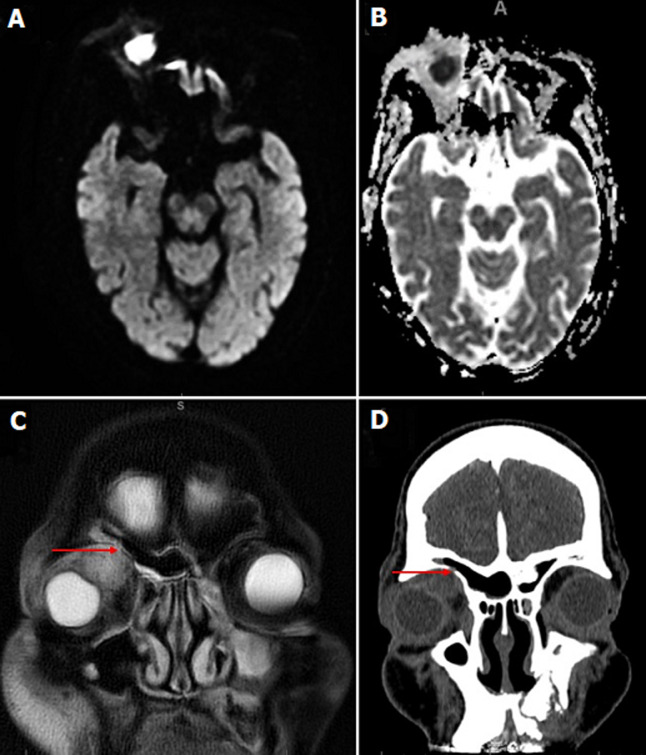
A) axial diffusion-weighted imaging MRI of the orbits without contrast; B) axial MRI image with companion apparent diffusion coefficient; C) coronal T2 fat suppressed MRI; D) coronal CT scan showing a superomedial orbital mass with restricted diffusion, and trace of fluid tracking to the frontal sinus (red arrow)

**Diagnostic assessment:** full blood count showed anemia with thrombocytopenia, inflammatory markers were elevated (CRP 43mg/L). Computerized tomography (CT) and magnetic resonance imaging (MRI) scan revealed a retro-orbital mass closely associated with the right lacrimal gland (Figure 2). Nasal endoscopy revealed a healthy right cavity with evidence of mucopus from the left middle meatus, consistent with his CT findings of left maxillary sinusitis.

**Figure 3:**
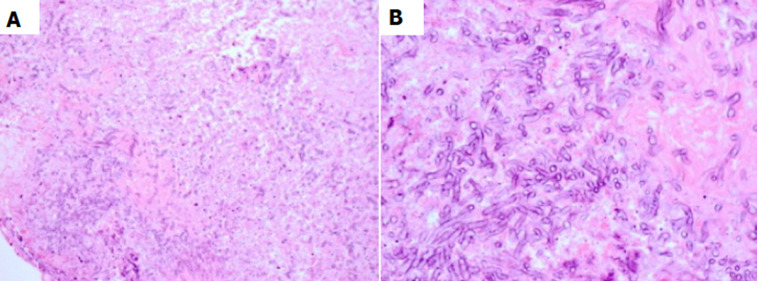
histological sections of orbital tissue biopsy showing fungal hyphae with septation; A) hematoxylin and eosin stain, x 200; B) hematoxylin and eosin stain, x 600

**Diagnosis:** he underwent an orbital biopsy revealing fungal hyphae with septation surrounded by chronic granulomatous inflammation, suggesting aspergillus species infection (Figure 3).

**Therapeutic interventions:** he received 10 days of intravenous (IV) amphotericin B, then switched to IV then oral voriconazole.

**Follow-up and outcome of interventions:** a repeat scan a month later showed an improvement in proptosis and slightly less bulky aspergilloma. He could not have contrast due to poor renal function. The MRI and CT scans were discussed at our local radiology meeting, and it was thought the orbital mass was communicating with the right frontal sinus (although there was only mild opacification). He developed deranged liver function tests as well as experiencing nightmares on voriconazole and this was discontinued after 6 weeks of treatment. A lower dose of voriconazole was restarted to avoid renal toxicity.

**Patient perspective:** the patient was initially anxious as he was unsure whether the swelling was related to his multiple myeloma. Once the swelling subsided, he felt reassured that his vision was unchanged. The treatment was the most challenging element, as he found the nightmares caused by the medication highly distressing, and was very happy when the course of antifungal medication was completed. To date, he has had no further recurrences.

**Informed consent:** yes, consent given to use images for educational purposes only (e.g. teaching, publications) and that all images will be cropped to only show the area of interest.

## Discussion

Aspergillus spores are frequent contaminants of the respiratory tract, rendering it the most likely cause of fungal infection of the paranasal sinuses. A ball of aspergillus (known as an aspergilloma) is typically found in the non-invasive type, but may become invasive in immunocompromised patients [2]. Sino-orbital aspergillosis carries high risk of mortality and morbidity due to potential central nervous system infection or subarachnoid hemorrhage secondary to ruptured mycotic aneurysms [3]. It is often misdiagnosed as patients present with variable symptoms and signs which, in turn, facilitates disease progression [4]. Treatment is typically poorly tolerated and the mortality rate remains high, despite appropriate therapy.

In our case report, the patient was immunocompromised with T2DM, multiple myeloma and CKD. He was initially thought to have a neoplastic lesion due to the MRI images and recent weight loss. Aspergilloma was diagnosed via histopathological examination of the biopsy. The origin of the infection was unclear. We postulate it arose from the frontal sinus due its location, mild frontal recess opacification on MRI scan, and possible communication. A limitation associated with this case report is that a sinus biopsy was not taken during endoscopy.

Sino-orbital aspergillosis most frequently arises from the maxillary sinus, followed by the ethmoid, sphenoid and rarely frontal sinuses [5-7]. The low susceptibility of the frontal sinus is likely secondary to the position of the ostium anterosuperior to the nasal cavity, making it less prone to inhaled spores [8]. Endoscopic sinus surgery for the frontal sinus is challenging due to the large anatomical variation of the frontal recess [9]; rendering the diagnosis more difficult.

Intraoperative findings of friable cheesy-like material that is green, yellow, brown or black are in keeping with a fungus ball. The diagnosis is typically confirmed by microscopic detection of fungal material, such as dichotomous, branching hyphae in smear. Debulking along with prolonged antifungal therapy can be effective in controlling the infection; in addition to retrobulbar amphotericin B injection [10]. Our case did not require exenteration unlike other reports [11]; this may be due to the more anterior location of the mass.

## Conclusion

Invasive sino-orbital aspergillosis is an aggressive disease and may be misdiagnosed as a tumor. We present a rare case likely arising from the frontal sinus. To improve prognosis, ophthalmologists should be aware of this distinct entity and use a multi-disciplinary approach.
